# Titanium-coated polypropylene mesh as innovative bioactive material in conservatives mastectomies and pre-pectoral breast reconstruction

**DOI:** 10.1016/j.bioactmat.2021.05.002

**Published:** 2021-05-19

**Authors:** Pietro Gentile, Marco Bernini, Lorenzo Orzalesi, Silvia Sordi, Icro Meattini, Francesca Lessi, Ashutosh Kothari, Claudio Calabrese

**Affiliations:** a Department of Surgical Science, University of Rome “Tor Vergata”, Rome, 00133, Italy; b Breast Surgery, Breast Unit, Oncology Department, Careggi University Hospital, 50134, Florence, Italy; c Department of Experimental and Clinical Biomedical Sciences “M. Serio”, University of Florence Radiation Oncology Unit - Oncology, Careggi University Hospital, Florence, Italy; d Fondazione Pisana per la Scienza onlus, Pisa 56122, Italy; e Breast Surgery Unit, Guy's Hospital, Guy's and St. Thomas' NHS Foundation Trust, London, United Kingdom; f San Rossore Breast Unit, Pisa 56122, Italy

**Keywords:** Mastectomy, Pre-pectoral breast reconstruction, Titanium-coated polypropylene mesh, Immediate breast reconstruction

## Abstract

Breast reconstruction is rapidly evolving, thanks to the growing acceptance of synthetic meshes as innovative biomaterials. 276 patients undergoing mastectomy (total of 328 mastectomies) were analyzed in a retrospective observational study to evaluate the pre-pectoral immediate breast reconstruction (IBR) using an implant wrapped with Titanium-Coated Polypropylene Mesh (TCPM) vs. patients treated with tissue expander (TE), equally placed pre-pectorally (and wrapped with the same TCPM in 74.3% of the control group’ breasts). 163 patients, of the study group (SG), underwent mastectomy and pre-pectoral IBR with implant wrapped with TCPM, in a one-step surgery, called direct-to-implant technique (DTI), while 113 patients control group (CG) underwent mastectomy and TE. DTI technique has been performed in 192 breasts of the SG while TE procedure in 136 breasts of the CG. The BREAST-Q questionnaire has been provided before the treatment and 2 years later. Baker scale has been used to evaluate capsular contracture. Oncologic, surgical, and aesthetic outcomes along with BREAST-Q scores were analyzed. Additionally, a histologic evaluation was conducted in 11 capsules' samples randomly chosen (6 derived from SG patients and 5 derived from CG). Complications were recorded in 43 cases (29SG-14CG): 8 skin-nipple necrosis (5SG-3CG), 8 wound dehiscence (6SG-2CG), 3 hematomas (1SG-2CG), and 24 infections (8SG-16CG). Grade IV capsular contracture was detected in 9 breasts (1SG-8CG), whereas 254 breasts were grade I (110SG-144CG), 33 (10SG-23CG) grade II, and 32 (4SG-28CG) grade III. Implant wrinkling was detected in 18 cases (10SG-8CG) after 30 months. The local tumor recurrence rate was 5.8%. Three recurrences were on the nipple-areola complex (1.9%). SG patients showed significantly higher rates in the BREAST-Q overall Satisfaction with Outcome (74.1), overall Satisfaction with Breasts (69.1), Psychosocial Well-being (81.9), and Sexual Well-being (63.1), versus CG's patients (p < 0.05). Histological analysis showed a process of normal tissue repair with a complete mesh integration and normal healing. Conservative mastectomies with pre-pectoral IBR assisted by TCPM proved themselves oncologically safe, biologically integrated into native tissues, and highly accepted in terms of quality of life guaranteeing a more natural and aesthetic breast appearance.

**Core tip:**

This retrospective observational study provided clinical and histological outcomes of the pre-pectoral IBR using an implant wrapped with TCPM vs. patients treated with TE, equally placed pre-pectorally. The efficacy of IBR using an implant wrapped with TCPM was confirmed by the cosmetic results obtained and by a rate of side effects comparable to TE. All the histological analyses performed confirmed the TCPM mesh complete integration with the physiological aspects of healing: The Collagen 1 and 3 expressions did not differ, between TCPM and NO TCPM samples to confirm a process of healing overlapping to perfect device incorporation and normal healing.

## Highlights

• This retrospective observational study, reports the results of pre-pectoral immediate breast reconstruction (IBR) using a prosthesis wrapped with Titanium-Coated Polypropylene Mesh (TCPM) vs. patients treated with tissue expander (TE) equally placed pre-pectorally (and wrapped with the same TCPM in 74% of the control group), with the aim to reduce the risk of reconstruction failure after conservative mastectomy.
• The primary end-points of the study were the clinical outcomes. These outcomes were: the incidence of perioperative and postoperative complications (safety profile), quality of life (QoL) at 2 years after surgery, measured as the change between the pre- and post-op BREAST-Q scores, the aesthetic outcomes, and the capsular contracture grade. Two methods for the clinical analysis of the results have been used: Surgical-Team and patient self-evaluation. The Surgical-team evaluation was an objective evaluation based on clinical assessment, using a scale of six degrees (excellent, good, discreet, enough, poor, inadequate). The subjective patient-based self-evaluation applied the same six degrees. Moreover, secondary end-point of the study, was a histopathological capsule assessment to evaluate the integration of the TCPM in the mastectomy flap as a bioactive material.
• Histological, and bio-molecular evaluation of TCPM samples were analyzed. In particular, Hematoxylin-eosin staining of post-operative biopsies of wrapping tissue TCPM mesh was performed focusing on the collagen presence and fibroblasts amount and specifically, on the complete healing and TCPM incorporation without side effects. Additionally, the immunofluorescence using specific markers CD 45 and Collagen 1, and immunohistochemistry using CD 45 on paraffin samples were performed. The endpoint was to evaluate the grade of inflammation represented by the number of lymphocytes in the treated site, and the eventual different collagen type expression (Collagen 1 and Collagen 3) in tissues underwent and not to radiotherapy and during the use of TCPM.
• Analyzing the side effects with the use of t-test, the complication rate between SG and CG did not showed statistically significant differences (p=0,8472), although it was slightly higher in the SG. Despite the study group was bigger than the control group (+42%) the side effects' number did not statistically differ, confirming the safety and reliability of the TCPM procedure during DTI. Overall Satisfaction with Breasts, Psychosocial Well-being, and Sexual Well-being scores were all significantly increased after sub-cutaneous pre-pectoral DTI immediate reconstruction with TCPM mesh (p < 0.05) compared to CG. Mean satisfaction with the overall aesthetic result was high after breast reconstruction (8.72) in patients who underwent DTI pre-pectoral immediate reconstruction (SG) after 2 years from surgery.
• The histological analysis of fragments of implant's wrapping tissue incorporating TCPM mesh displayed complete incorporation of the mesh with physiological aspects of healing. The immunochemistry concluded the Collagen 1, and 3 expressions did not differ, with statistical significance, between TCPM and NO TCPM samples, showing a process of physiological healing overlapping to perfect device incorporation, confirming that TCPM was a bioactive material. Conservative mastectomies with pre-pectoral IBR assisted by TCPM are resulted oncologically safe preserving breast appearance.

## Graphical abstract

**Figure:**
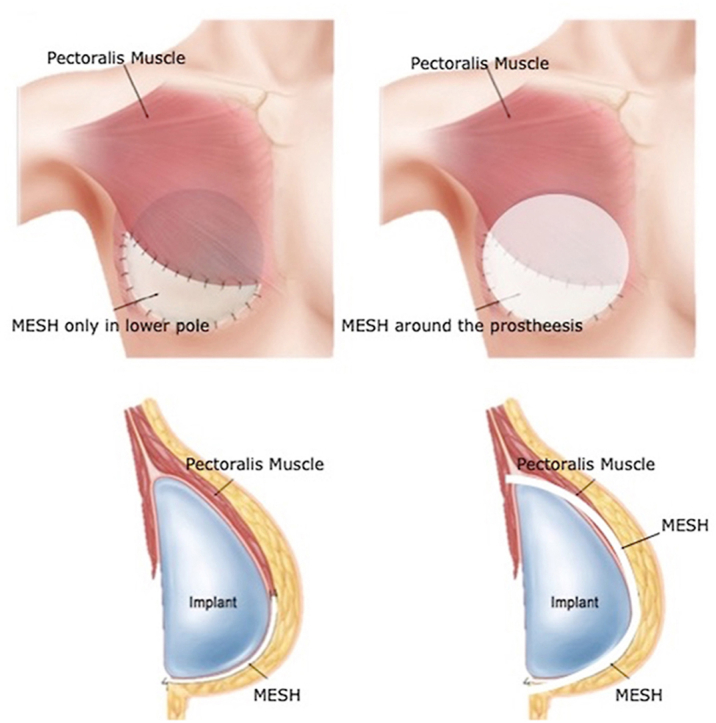

## 1 Introduction

Skin-Sparing Mastectomy (SSM) or Skin-Reducing Mastectomy (SRM) and Nipple-Sparing Mastectomy (NSM), often called conservative mastectomies, can be all considered oncoplastic surgery developments born with Veronesi U [1]. Meta-analyses investigations displayed that SSM and NSM results do not differ from those for non-conservative mastectomies [2]. Recurrence rates in the nipple-areola complex (NAC) after NSM are acceptably low (0–3.7%) [2], additionally the psychological adjustment and patient satisfaction rate are good in people who underwent NSM [3]. The principal side effect of NSM is NAC necrosis, and factors promoting it are large breasts, breast ptosis, radiotherapy, smoking, and obesity [2,3]. For patients needing mastectomy, NSM should be the first choice [3].

Carlson et al. [4] reported 4 different types of SSMs: types I to III for a small breast with a low ptosis degree using a peri-areolar approach; type IV for a large breast with high ptosis degree plus breast reduction of the contralateral breast. The introduction of the SRM concept, based on permanent implant positioning into a large pocket made by the pectoralis major muscle and an inferior pedicle dermal flap, allows obtaining a safe oncologic result with a cosmetically satisfying reconstruction [5]. Conservative mastectomies entail both cancer removal and preparation of skin flaps, usually allowing an immediate breast reconstruction (IBR) and breast reshaping to provide better aesthetic outcomes, without compromising local disease control [6]. The most important advantage of IBR is to avoid the necessity of repeated several surgical procedures to restore breast profile.

Breast reconstruction can be based on autologous tissue or prosthesis which could be positioned immediately or in two stages (tissue expander and prosthesis). Different techniques of breast implant positioning (subcutaneous, sub-muscular, dual-plane) were pioneered in the last decades.

Snyderman and Guthrie [7] in 1971 reported the subcutaneous placement of implants as the first attempt in prosthetic reconstruction. This procedure, however, was associated with a high rate of side effects, such as mastectomy skin flap necrosis, prosthesis extrusion, and capsular contracture [8,9]. Subsequently, Gruber et al. [10] and Argenta et al. [11] reported superiority of a reconstruction performed with a sub-muscular prosthesis (described as retro- or sub-pectoral) over the subcutaneous to improve the aesthetic outcomes and reducing the side effects rate [10,11]. As the sub-pectoral pocket left after mastectomy was assumed to be too small to accommodate an implant, many surgeons opted, in the past, for the two-stage sub-muscular implant-based breast reconstruction.

The introduction of biomaterials as biological and/or synthetic matrices opened new scenarios in prosthetic-based breast reconstruction (PBBR). A combined partly sub-muscular and partly sub-matrix dual-plane implant pocket has now become the standard of care. These matrices provide the opportunity of enlarging the sub-muscular pocket thus allowing adequate prosthesis coverage for a definitive PBBR in one-step surgery, the so-called direct-to-implant technique (DTI) [[12], [13], [14], [15], [16], [17], [18]].

The idea of totally wrapping the implant with these matrices acting as bioactive materials, resurrected the concept of a total pre-pectoral approach in breast reconstruction, allowing a more natural prosthesis positioning in the subcutaneous plane [[14], [15], [16], [17], [18]]. This procedure was introduced in 2014 positioning the prosthesis in a subcutaneous pocket, wrapped by a titanium-coated polypropylene mesh (TCPM) [10]. Additionally, two investigations showed the preliminary outcomes of a pre-pectoral DTI reconstruction, with prosthesis wrapped by a biological a-cellular dermal matrix (BADM) [16,17].

A recent study of Casella et al. [19] displayed the long-term results of TCPM, (wrap-around technique after mastectomy and sub-cutaneous DTI breast reconstruction), reporting the BREAST-Q evaluation.

The rationale for the present investigation and of a pre-pectoral reconstruction performed with TCPM is this device may replicate in a sub-cutaneous approach, the same coverage effect of muscles in the retro-pectoral technique, without any muscular detachment. The authors' effort has been to confirm the assumption that a sub-cutaneous - pre-pectoral reconstruction using soft tissue replacement bioactive materials, as TCPM mesh, is feasible, safe, and giving more rewarding outcomes particularly when it is done in a one-step (DTI) approach. Moreover, a biomaterial is considered bioactive when it is able to integrate into the host tissue through physiological and controlled reactions.

Therefore, the aim of the present work was also to demonstrate, through the histological analysis supported by immunofluorescence and immunochemistry, the physiological incorporation of the synthetic TCPM mesh after pre-pectoral immediate breast reconstruction.

## 2 Methods

### 2.1 Study overview and data definitions

This observational case-controlled study was conducted following the principles outlined in the Declaration of Helsinki, as revised in Tokyo in 2004 and internationally consented ethics in clinical research [20]. A quality assessment was carried out based on the STrengthening the Reporting of Observational Studies in Epidemiology (STROBE) checklist [21]. The study protocol was previously approved in 2011, as already reported [15,19], by the Institutional Ethics on Research Committee of the University-Hospital, “Careggi” Florence, Italy. Additionally, the protocol has been performed following the European rules 726/2004/EC, 2001/83/EC, 2001/82/EC, and 2001/20/EC. Also, the study was subject matter of a research contract (R.D. #1467/2017) and associate professor contract (R.D. #13489/2021) between the first author P.G., and the University of Rome, “Tor Vergata”. All patients signed an informed consent form, before any surgical treatment, in which detailed information about the study, including the risks, benefits, and alternative therapies, has been reported.

### 2.2 Patient population

**Scheme 1:**
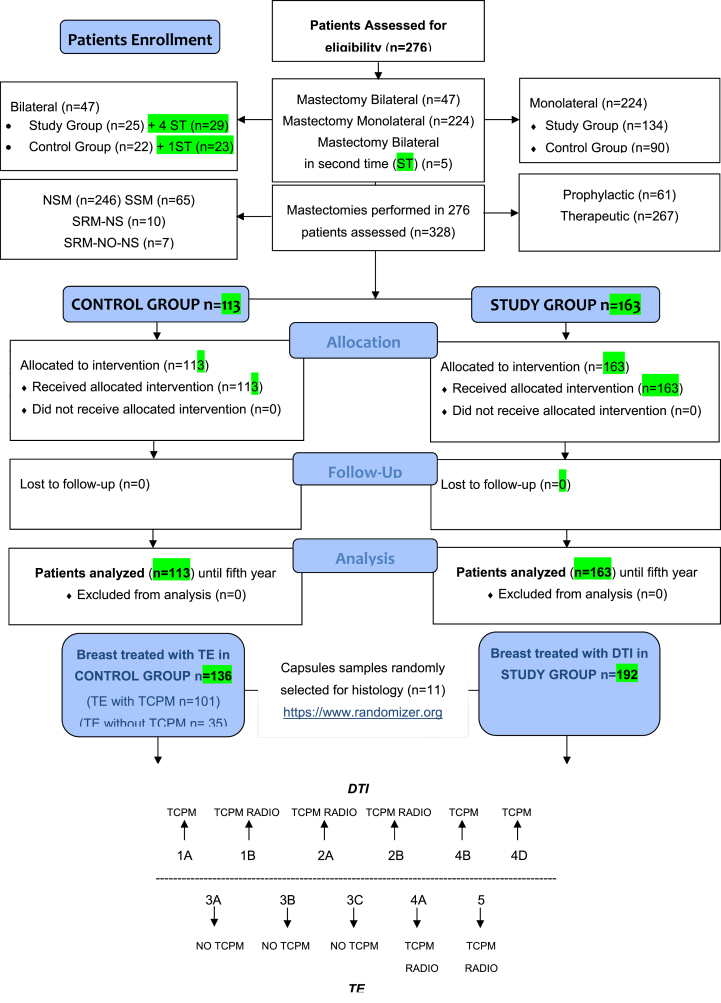
CONSORT (Consolidated Standards of Reporting Trials) flow diagram on patients' enrollment, including mastectomies and treatments assessment.

Between January 2012 and December 2018, 590 females undergoing mastectomy were enrolled, of which, 276 were assessed for eligibility. 328 interventions were performed in the assessed population. Patients and mastectomies assessment were shown in a CONSORT flow diagram (Scheme 1).

Inclusion and exclusion criteria were considered. The inclusion criteria were indication to NSM, SSM and SRM either therapeutic or prophylactic, age 25–85 years old, and BMI 16–40. On the other hand, exclusion criteria were T4 and metastatic cancer, refusal to sign the specific informed consent, uncontrolled comorbidities or multiple comorbidities (three or more among diabetes, renal failure, heart failure, cardiovascular diseases, hypertension, pulmonary diseases, hepatic diseases and metabolic diseases). The presence of only one or two comorbidities (in particular diabetes and/or hypertension), were not considered exclusion criteria. Smoking, previous radiotherapy and chemotherapy were not considered exclusion criteria. Equally, previous breast surgery was not considered exclusion criteria.

All enrolled patients were undergoing a full preoperative screening, including a complete clinical examination, photographic, and cancer assessments. Before surgery, all patients were evaluated for both autologous or alloplastic breast reconstruction, taking into account patient preference, body habitus, comorbidities, and surgeon experience. Baseline characteristics were listed in Table 1 while Table *2* listed the characteristics of the surgical procedures.

**Table 1:** Baseline characteristics of the patients included in the analysis.

Characteristics		Patients n = 276	Percentage %	SG	CG
**Age**	Mean	55		54	56
	SD	**±**10.33		**±**10.11	**±**10.55
	Range	(26–84)		(26–82)	(28–84)
**BMI**	Mean	28		27	29
	SD	**±**4		**±**3.85	**±**4.25
	Range	(16–40)		(16–38)	(18–40)
**Smoke**	No	231	83.7%	133	98
	Current	45	16.3%	26	19
**Hypertension**	No	228	82.6%	123	105
	Yes	48	17.4%	36	12
**BRCA mutation**	No	254	92.0%	149	105
	Yes	22	8.0%	10	12
**Diabetes**	No	268	97.1%	155	113
	Yes	8	2.9%	4	4
**Previous RT**	No	237	85.9%	137	100
	Yes	39	14.1%	22	17
**Mastectomy**	Bilateral	47	17.0%	28	19
	Bilateral in second time	5	1.8%	2	3
	Unilateral	224	81.2%	129	95

Post-mastectomy flaps were always evaluated as adequate, and no reconstruction was aborted. The conservative mastectomy with sub-cutaneous DTI pre-pectoral reconstruction in one-step surgery, assisted by TCPM mesh, has been performed in 163 patients -Study Group- (SG), treating 192 breasts (58.5%) while TE pre-pectoral procedure has been done in 113 patients -Control Group- (CG), treating.

136 breasts (41.5%), followed by a second step represented by the tissue expander changing with a definitive implant. Breasts of the CG patients were divided into two subgroups: CG′ breasts treated using TE wrapped with TCPM (n = 101) and CG’ breasts who underwent TE without TCPM (n = 35).

The size of the implants used both during DTI and after TE changing with definitive prostheses was between 270 and 470 cc, while the size of the TE was between 250 and 500 cc. The choice of the definitive prosthesis (both anatomical and round) was performed based on the tissues available, with “custom made” methods.

The average tumor size was 31.8 mm (range, 0–82 mm). The drain was removed between the second and ninth postoperative days (mean, 4.2 days). The definitive pathology report was invasive ductal carcinoma in 153 cases, ductal carcinoma in situ in 23 cases, invasive lobular carcinoma in 6 cases, and ductal carcinoma in situ with foci of invasive ductal carcinoma in the remaining cases. Tumor-related data are summarized in Table 2.

**Table 2:** Baseline characteristics of interventions included in the analysis.

		Interventions n = 328	Percentage %
**Mastectomy**	Prophylactic	61	18.6%
	Therapeutic	267	81.4%
**Mastectomy**	NSM	229	69.8%
	NSM-modified “T-inverted”	17	5.2%
	SRM - Nipple Sparing	10	3.0%
	SRM- No Nipple Sparing	7	2.1%
	SSM	65	19.8%
**Previous Surgery**	1 = Wide Excision Unilateral	34	10.4%
	2 = Wide Excision Contralateral	5	1.5%
	4 = QUART Unilateral	34	10.4%
	5 = QUART Contralateral	7	2.1%
	6 = Breast Augmentation	7	2.1%
	7 = Contralateral Mastectomy	22	6.7%
	8 = None	219	66.8%
**Axilla**	SNB Final	102	31.1%
	SNB Intraoperative Negative	77	23.5%
	SNB + Axillary Dissection	22	6.7%
	SNB + Axillary Dissection Delayed	15	4.6%
	Axillary Dissection	37	11.3%
	No	75	22.9%
**Cutting**	Linear	59	18.0%
	Periareolar	21	6.4%
	S in External Quadrants	200	61.0%
	Inframammary Fold	10	3.0%
	Inverted T	36	11.0%
	Vertical	2	0.6%
**Radiotherapy**	No adjuvant	278	85.0%
	Yes adjuvant	49	15.0%
**Chemotherapy**	Adjuvant	93	28.4%
	Neo-adjuvant	30	9.1%
	No	205	62.5%
**Hormone Therapy**	No	138	42.2
	Yes	189	57.8
**Trastuzumab**	No	277	84.7
	Yes	50	15.3
**Device**	Prosthesis (DTI)	192	58.5
	Expander (TE)	136	41.5
**pT**	T0	79	24.1
	Tis	44	13.4
	T1	156	47.6
	T2	47	14.3
	T3	2	0.6
**pN**	N0-X	251	76.4
	N1	52	15.9
	N2	13	4.0
	N3	12	3.7
**ER**	–	30	9.1
	+	214	65.2
	Not performed	84	25.6
**PgR**	–	52	15.9
	+	192	58.5
	Not performed	84	25.6
**Ki67**	<20%	79	24.1
	>20%	130	36.9
	Not performed	119	36.3
**Her 2**	Amplified	51	15.5
**Lymph-nodes invasion**	Absent	145	44.2
	Present	56	17.1
	Missing	127	38.7

The choice to perform a DTI reconstruction or a TE reconstruction has been done by the single-center Multi-Disciplinary Team (MDT)'s oncological and reconstructive guidelines and determined by the patient characteristics and preferences. Thin patients, with emaciated and stretched skin were not considered eligible candidates to DTI. Equally obese, hypertensive and diabetic patients and smokers were not considered good candidates as well, due to of a compromised microvascular performance of their skin flap. The features of treated patients were reported in Table 1.

Post-operative follow-up was scheduled at 2, 7, 15, 21, and 36 weeks and then annually for five years.

### 2.3 Clinical data assessment

Data have been prospectively sign-in a database using SQTM® software (CPO, Turin, Italy), an application designed for the breast cancer therapy’ quality control. The following characteristics were prospectively recorded in the dataset: demographic data, age, BMI, histological evaluation, surgical and oncological management, surgical complications, time and site of recurrence, adjuvant or previous radiotherapy, and chemotherapy data. All the therapeutics options were discussed and decided by an MDT, including a breast surgeon, a plastic surgeon, a pathologist, a radiologist, an oncologist, a radiotherapist, and a psycho-oncologist.

During the first five years, patients were followed up every six months by clinical examination and oncological examinations as required and every twelve months by surveillance mammogram and ultrasound (US) or magnetic resonance imaging (MRI) if needed. Recurrences were documented by clinical examination, radiological tests, and pathological assessment. Local and distant recurrence rates evaluation were additional end-points of the study and were evaluated as the oncological safety outcome.

The primary end-points of the study were the clinical outcomes. They were: the incidence of perioperative and postoperative complications (safety profile), quality of life (QoL) at 2 years after surgery, measured as the change between the pre-operative and post-operative BREAST-Q scores (questionnaire on Satisfaction with Breasts, Satisfaction with Outcome, Psychosocial Well-being, Physical Well-being, and Sexual Well-being, post-operative questionnaire was administered after second-stage surgery in case of TE reconstruction), the aesthetic outcome (assessed by an independent panel of plastic surgeons, based on standardized photographs taken two years after surgery in DTI cases and after two years from second stage in TE cases) and the capsular contracture grade, assessed by a panel of plastic surgeon through outpatient clinic visits.

The Surgical-team aesthetic evaluation was an objective evaluation based on clinical assessment, using a scale of six degrees (excellent, good, discreet, enough, poor, inadequate).

At every evaluation degree was assigned a single numeric unit score to be summed-up for a final score for each patient considering the following variables: breast volume, shape, position of infra-mammary fold, and scars.

All adverse events were recorded for two years after breast reconstruction. Surgical complications were classified as those requiring a surgical re-treatment, including skin-nipple necrosis, seroma, wound dehiscence, wound infection, and hematoma. Such reinterventions consisted in a revision surgery when possible or in the implant/TE removal when necessary (failure cases). Other surgical interventions performed purely for aesthetic purposes were considered separately in the statistical analysis. The Baker scale was used for scoring capsular contracture during postoperative follow-up.

Moreover, as a secondary end-point of the study, a histopathological assessment of the capsule was performed, to evaluate the integration of the TCPM in the mastectomy flap as a bio-active material.

### 2.4 Surgical protocol

**Fig. 1:**
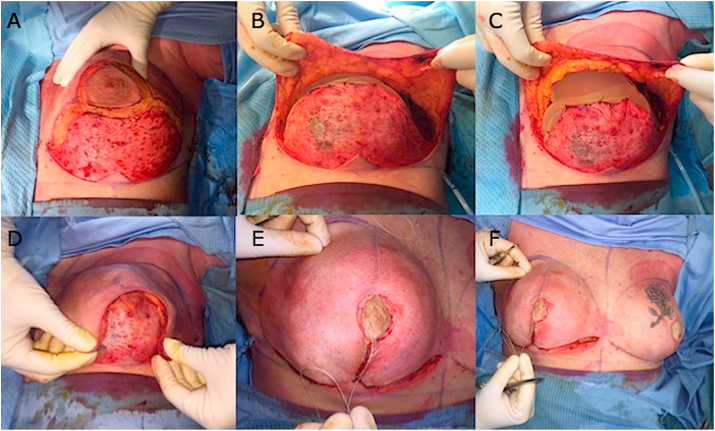
Intra-surgical view of a bilateral SRM-Nipple Sparing, with TCPM mesh use, and re-grafting of NAC according to Thorek technique. A) Surgical incision during SRM according to Wise pattern. B) Dermal-skin flap preparation. C) Prosthesis placement under the TCPM mesh. D) Skin flap positioning down over the prosthesis and mesh. E) NAC re-implanting as a free graft according to the Thorek technique and its fixation over the skin flap. F) Final sutures and contralateral identical procedure in a bilateral SRM for a BRCA mutation in a very large and ptotic breast. Clinical cases and outcomes were shown in Fig. 2.

Surgical incisions (Fig. 1A, representing an SRM-NS) were done according to the surgeon's experience, his preference, and to the type of conservative mastectomy chosen. Sentinel lymph-node/s biopsy and axillary surgery, when needed, were performed according to the single oncological requirements. The choice of SSM, NSM, or SRM mastectomy was carried out following the surgical oncology criteria and according to breast volume and ptosis. Medium or small breast (cup size A–B, 200–500 g) with < 8 cm of NAC-inframammary fold distance, no more than grade 1 ptosis according to the Regnault classification, patients' desire of breast reconstruction with a volume no larger than the preoperative one, were considered selective criteria to perform a DTI after NSM. A pre-operative evaluation of the subcutaneous breast tissue or “gland envelope” thickness, represented by the distance between the skin and the gland through a digital mammogram has been performed. Pre-operative digital mammogram, as suggested by Rancati et al. [41], allows an accurate evaluation of the breast coverage and a preview of the resulting flap thickness, with a consequent possible prevision of flap quality and vascularization. Therefore, the patients' selection candidate to DTI has been additional performed favoring those with breast subcutaneous tissue coverage above 2 cm at pre-operative digital mammography (type 3 according to Rancati score [41]), as to obtain an adequate flap after NSM, represented by the distance between the superficial layer of the fascia superficialis (the mastectomy “safe” surgical plane) and the skin. At the end of mastectomy, skin flap viability (Fig. 1B) and the confirmation of a sub-cutaneous pre-pectoral reconstructive approach were left to the evaluation of the operating oncoplastic surgeon. Muscles, pectoralis major and serratus, were not dissected at all in both DTI procedures and TE reconstructions. Partial resection of pectoralis major muscle was allowed, as needed, only if cancer's infiltration was confirmed.

### 2.5 Breast reconstruction options: DTI reconstruction using prosthesis and mesh

During DTI reconstruction, the TCPM (Fig. 1C), here called “supportive material” and the prosthesis were prepared for implantation via two different modes: the prosthesis was completely wrapped by the TCPM or, it was covered only on its anterior profile. In the first case, a purse-string suture on three sides of the supportive material sheets was adopted to create a “bag” around the prosthesis itself. In the case of larger implants, two TCPM sheets were sutured together along three sides to create a pocket where the implant will have been positioned from the opening. The supportive material will have to be tightly enfolded around the prosthesis with just a few loose ends of excessive material on the upper border.

After being washed with an antibiotic solution of Gentamicin, the implant and the TCPM were inserted on the mastectomy site. Once the appropriate orientation of the prosthesis had been verified, TCPM was secured using two interrupted stitches between the muscular fascia and the upper border of the supportive material, one on the sternal edge and the other one on the axillary anterior line.

In the second case, the prosthesis was covered only on its anterior profile. In such circumstances TCPM was transferred on the mastectomy site and secured to the muscular fascia, with few interrupted stitches, to cover the upper border of the prosthesis in first place. Afterward, the implant was placed under the supportive material (Fig. 1C), which, like a tent, was stretched to tightly cover the implant. With everything in place, the operator secured the inferior border of the supportive material, with few interrupted stitches, to keep it tightly folded around the prosthesis lower pole. At this point, the skin flap was stretched down (Fig. 1D), and a free graft NAC repositioning was performed (Fig. 1E). Contralateral breast mastoplasty (either augmentation, mastopexy or reduction mastoplasty) was performed where indicated or a bilateral identical procedure was performed in case of bilateral mastectomy (Fig. 1F).

### 2.6 Breast reconstruction options: TE reconstruction in combining with mesh

During the TE-reconstruction based on two steps, once a right size expander was chosen, the TE was prepared with the supportive material TCPM in two different ways, as previously described for DTI: the TE was completely wrapped by TCPM or it was covered only on its anterior profile. The wrapping procedures of TCPM may be considered as totally overlapping to those of DTI. In the case of larger expanders, as in larger prostheses during DTI, two TCPM sheets were sutured together along three sides to create a pocket where the implant will be positioned from the opening. When wrapping a TE, differently to DTI, the supportive material will have to be loosely enfolded around the expander with enough space left for future expansions, even though using a re-absorbable suture to fix the mesh an easy expansion of the mesh itself along with the TE is guaranteed after few weeks. During the second step of TE exchange for the implant, a moderate capsulotomy was performed only where necessary, but a complete capsulectomy was not permitted, being the integrated supportive material an essential cornerstone of the pre-pectoral approach, recreating a new fascia.

For each patient, both SG and CG, the surgical incision was sutured after excising a 2 mm wide skin stripe on both sides of the incision itself. At least two layers of sutures were performed. 3–0, 4–0, and even 5-0 monofilament re-absorbable sutures were applied depending on the surgeon's preference. Both interrupted and running sutures were allowed.

### 2.7 Clinical evaluation of aesthetic outcomes: primary endpoints

Two methods for the clinical analysis of the results, primary endpoints of the study have been used: Surgical-Team-evaluation and patient self-evaluation. The Surgical-team evaluation was an objective evaluation based on clinical assessment, using a scale of six degrees (excellent, good, discreet, enough, poor, inadequate). The subjective patient-based self-evaluation applied the same six degrees. Breast volume, breast shape, position of infra-mammary fold, and scars were evaluated.

### 2.8 Histological evaluation and secondary endpoints

As a secondary endpoint of the present study, a histological assessment was conducted to evaluate the outcomes of TCPM mesh tissue integration both in DTI and TE procedures, with biopsies taken intraoperatively during reinterventions for SG cases and second-stage operations for CG. Hematoxylin-eosin staining of post-operative biopsies of wrapping tissue TCPM mesh was performed focusing on the collagen presence and fibroblasts amount and specifically, on the complete healing and TCPM incorporation without side effects.

Additionally, the immunofluorescence using specific markers CD 45 and Collagen 1, and immunohistochemistry using CD 45 on paraffin samples were performed.

The endpoint was to evaluate the grade of inflammation represented by the number of lymphocytes in the treated site, and the eventual different collagen type expression (Collagen 1 and Collagen 3) in tissues which underwent or not to radiotherapy and during or not the use of TCPM.

### 2.9 Histological and bio-molecular analyses of mastectomy flap biopsies

The biopsies of the mastectomy flaps previously undergone to sub-cutaneous pre-pectoral DTI and TE reconstruction were obtained from 11 patients randomly chosen (called 1A, 1B, 2A, 2B, 3A, 3B, 3C, 4A, 4B, 4D, 5) (Scheme 1), six derived from SG patients who underwent DTI with TCPM mesh (three treated with radiotherapy and three radio free), and five derived from CG patients treated with TE (two treated with TCPM and radiotherapy and three not treated with TCPM and radio free). Biopsies were all performed in a time span between 12 and 24 months after the surgical procedures. The punch biopsies were all performed during a further surgical procedure under general anesthesia at the moment of TE exchanging with definitive implants in CG (two-stage reconstructions), while they were performed during a second operation in SG's patients who underwent DTI. The anatomical part of the breast chosen to be punched was different according to the type of mastectomy performed, and the skin incision but in each case, it was performed in the proximity of the scars (to obtain invisible outcomes) harvesting samples of neo-fascia. Antibiotic prophylaxis was performed in all cases. 11-*μ*m paraffin sections were stained with Hematoxylin and Eosin for histological analysis. Three slides for each patient were carefully analyzed 10^x^ and 40^x^ high power fields to investigate the number of lymphocytes, collagen, and fibroblast amounts. In parallel, immunofluorescence was introduced to evaluate the different Collagen 1 and 3 expressions inside all of the tissue undergone TCPM ± radiotherapy and in tissue free from TCPM ± radiotherapy.

A scoring system was elaborated to quantify the percentage of Collagen 1 and Collagen 3. A score of 200, 400, 600, 800, or 1000 was assigned to the area that was covered by each type of collagen identified as a proportion of the entire area of every single slide analyzed. Subsequently, the average score of all slides for the eleven patients was calculated.

### 2.10 Patient selection for capsule biopsy

The patient's choice, relating to the biopsy selection, was created with the use of an online randomization generator (https://www.randomizer.org) among all the SG and CG patients undergoing a second operation after mastectomy and turned into concealed entities through someone unrelated to the trial. The participant histology assessors were all blinded to the type of surgery, and blinding was maintained until all information had been analyzed.

### 2.11 Statistical analysis

Using the Q Score Scoring Software, BREAST-Q scores were converted from survey raw scores (1 through 4 or 5) to a continuous range from 0 to 100, with a higher score representing greater satisfaction or better health-related quality of life. The scores for each BREAST-Q matrix index were determined at each time point and then entered into the database, along with the other data collected from patients and medical records. Both absolute BREAST-Q scores and their changes before and after treatment were analyzed. The Shapiro-Wilk test was used to verify for normal distribution of continuous variables. Consequently, BREAST-Q scores and panel scores were analyzed as continuous variables using the *t*-test. Values of *p* < 0.05 were considered statistically significant. A comparison of clinical and biological characteristics between the patients was performed by the Kruskal Wallis rank test for continuous variables and Pearson's Chi-square test (or Fisher's exact test when appropriate) for categorical variables. Logistic regression was used to investigate which factors were associated with recurrence events, either loco-regional or systemic. Independent variables of interest were surgical technique, age above 50 years old, lymph-vascular invasion, oncological stage, adjuvant or neoadjuvant chemotherapy, adjuvant radiotherapy, and adjuvant hormone therapy. Multiple logistic regression analyses were performed to account for several confounding variables simultaneously and included all variables of interest.

## 3 Results

### 3.1 Safety and oncological outcomes

The average post-surgical follow-up period was 44 months (range, 23–65 months). Complications were recorded in only 43 cases (13.1%) of which 29 cases (67.4%) belonged to the SG and 14 cases (32.6%) to the CG. To have a better comprehension of the outcomes it is necessary to consider that the SG comprised 159 patients, while the CG 112. In this way, the percentage of complications in SG was 18,3% versus 12,5% of the CG, where the SG was bigger by 42% than the CG (47 patients more versus CG).

These complications recorded in 43 cases were:

- Skin-nipple necrosis (8 cases), of which 5 in SG and 3 in CG;
- Wound dehiscence (8 cases), of which 6 in SG and 2 in CG;
- Hematoma (3 cases), of which 1 in SG and 2 in CG;
- Infection (24 cases), of which 8 in SG and 16 in CG.

Analyzing the side effects with the use of *t*-test, the complication rate between SG and CG did not shown statistically significant differences (*p* = 0,8472), although it was slightly higher in the SG.

Despite the study group being bigger than the control group (+42%) the side effects' number did not statistically differ, confirming the safety and reliability of the TCPM procedure during DTI.

A second operation was performed in all of these cases, and in 15 cases implant/TE removal was required (4.6%), followed by reconstruction with sub-muscular expanders in all cases. In 7 cases of infection, the authors removed only the implant without removing the wrapped-around matrix pocket, successfully clearing the infection. 5 patients who underwent prophylactic NSM and DTI reconstruction had positive ductal carcinoma in situ, in the retro-areolar tissue, at final histology. In these cases, the excision of the NAC was performed; none of these patients experienced the loss of the implant, and they all maintained the reconstructed breast. Safety profile and oncologic outcomes are summarized in Table 3.

**Table 3:** Safety profile and oncologic outcomes.

		N = 328	Percentage %	SG	CG	Percentage %
Early Complications	No	285	86.9%	163	122	57.2% (SG) - 42.8% (CG)
	Yes	43	13.1%	29	14	67.4% (SG) - 32.6% (CG)
	Wound Dehiscence	8	2.4%	5	3	62.5% (SG) - 37.5% (CG)
	Hematoma	3	0.9%	1	2	33.3% (SG) - 66.7% (CG)
	Infection	24	7.3%	8	16	33.3% (SG) - 66.7% (CG)
	Skin-nipple necrosis	8	2.4%	5	3	62.5% (SG) - 37.5% (CG)
Implant Removal	No	313	95.4	185	128	59.1% (SG) - 40.9% (CG)
	Yes	15	4.6	7	8	46.7% (SG) - 53.3% (CG)
Symmetrisation	Immediate	14	4.3%	8	6	57.1% (SG) - 42.9% (CG)
	Delayed	36	11.0%	18	18	50.0% (SG) - 50.0% (CG)
	No	174	53.0%	99	75	56.9% (SG) - 43.1% (CG)
	Bilateral Mastectomy	104	31.7%	42	62	40.4% (SG) - 59.6% (CG)
Second Look	Fat Grafting (1)	53	16.2%	33	20	62.3% (SG) - 37.7% (CG)
	Fat Grafting (2)	7	2.1%	1	6	14.3% (SG) - 85.7% (CG)
	Fat Grafting (3)	1	0.3%	0	1	0% (SG) - 100% (CG)
	Implant Exchange	1	0.3%	0	1	0% (SG) - 100% (CG)
	Implant Exchange + Fat Grafting	3	0.9%	1	2	33.3% (SG) - 66.7% (CG)
	No Fat Grafting	257	78.4%	134	123	52.1% (SG) - 47.9% (CG)
	NAC Reconstruction + Fat Grafting	6	1.8%	2	4	33.3% (SG) - 66.7% (CG)
Capsular contracture	Grade IV	9	2.7%	1	8	11.1% (SG) - 88.9% (CG)
	Grade III	32	9.7%	4	28	12.5% (SG) - 87.5% (CG)
	Grade II	33	10%	10	23	30.3% (SG) - 69.7% (CG)
	Grade I	254	77.4%	110	144	43.3% (SG) - 56.7% (CG)

Two years after surgery, grade IV capsular contracture was detected in 9 breasts (1 in SG and 8 in CG), whereas 254 breasts were evaluated as grade I (110 in SG and 144 in CG), 33 breasts (10 in SG and 23 in CG) were evaluated as grade II, and 32 breasts (4 in SG and 28 in CG) were evaluated as grade III. The total rate of significant (Baker III to IV grade) capsular contracture was reported as low as 12.5%. Patients who reported grade IV capsular contracture had all undergone adjuvant radiotherapy in the post-operative period. In case of severe contracture, implant replacement was required and was performed after an average of 28 months. Implant wrinkling was detectable in 18 cases (10 in SG and 8 in CG) after 30 months, and two or three fat grafting procedures were performed, successfully reducing implant visibility. The average fat volume injected was 73.3 ml per breast in each fat grafting session. The local tumor recurrence rate was 5.8%. Three recurrences were on the NAC (1,9%), two recurrences in axillary lymph-nodes (1,3%), three recurrences in skin flap (1,9%) and one recurrence (0,7%) in the muscle. The median time of loco-regional recurrence appearance was 21 months (range, 8–37 months). All risk factors for early complications and risk factors for failures were reported in Table 4 and Table 5 respectively.

**Table 4:** Risk factors for early complications.

	Early complication cases (N = 43)	Cases without complication (N = 285)	OR (95% CI) *p*-value	Adjusted OR (95% CI) *p*-value
**Age > 55**	20 (46.5%)	78 (27.4%)	2.31 (1.20, 4.44)	1.36 (0.57, 3.22)
			0.012	0.488
**BMI > 25**	13 (30.2%)	70 (24.6%)	1.33 (0.66, 2.69)	0.69 (0.27, 1.73)
			0.426	0.427
**Smoke**	7 (16.3%)	48 (16.8%)	0.96 (0.40, 2.28)	1.61 (0.62, 4.19)
			0.927	0.333
**Diabetes**	7 (16.3%)	1 (0.3%)	55.22 (6.60, 461.8)	45.32 (4.35, 472.2)
			<0.001	0.001
**Hypertension**	13 (30.2%)	39 (13.7%)	2.73 (1.31, 5.69)	2.89 (1.03, 8.09)
			0.007	0.044
**Radio pre**	15 (34.9%)	33 (11.6%)	4.09 (1.98, 8.44)	7.16 (2.68, 19.13)
			<0.001	<0.001
**Radio post**	6 (13.9%)	44 (15.4%)	0.89 (0.35, 2.23)	1.59 (0.56, 4.48)
			0.801	0.384
**Chemo pre**	2 (4.7%)	28 (9.8%)	0.45 (0.10, 1.95)	0.90 (0.18, 4.39)
			0.285	0.894
**Chemo post**	11 (25.6%)	82 (28.8%)	0.85 (0.41, 1.77)	0.83 (0.34, 2.01)
			0.665	0.682
**Hormone therapy**	28 (65.1%)	161 (56.7%)	1.43 (0.73, 2.79)	1.40 (0.61, 3.21)
			0.299	0.423
**Device**				
DTI	**29 (67.4%)**	**163 (57.2%)**	1.48 (0.79, 2.81)	1.41 (0.63, 3.32)
			0.301	0.478
TE	**14 (32.6%)**	**122 (42.8%)**	0.64 (0.33, 1.27)	0.55 (0.24, 1.22)
			0.206	0.140
**Mastectomy**				
Prophylactic	5 (11.6%)	56 (19.7%)	0.79 (0.31, 2.10)	1.53 (0.43, 4.10)
			0.752	0.256
Therapeutic	38 (88.4%)	229 (80.3%))	1.86 (0.70, 4.94)	2.09 (0.65, 6.72)
			0.214	0.216

**Table 5:** Risk factors for failures.

	Failure cases (N = 15)	Cases without failure (N = 313)	OR (95% CI) p-value	Adjusted OR (95% CI) p-value
**Age > 55**	9 (60.0%)	89 (28.4%)	3.78 (1.31, 10.91)	2.66 (0.74, 9.51)
			0.014	0.132
**BMI > 25**	5 (33.3%)	78 (24.9%)	1.51 (0.50, 4.54)	0.76 (0.20, 2.85)
			0.467	0.681
**Smoke**	4 (26.7%)	51 (16.3%)	1.87 (0.57, 6.10)	2.52 (0.67, 9.53)
			0.301	0.173
**Diabetes**	0 (0.0%)	8 (2.6%)	Omitted	Omitted
**Hypertension**	6 (40.0%)	46 (14.7%)	3.87 (1.31, 11.39)	3.96 (1.01, 15.63)
			0.014	0.050
**Radio pre**	3 (20.0%)	45 (14.4%)	1.49 (0.40, 5.48)	3.54 (0.73, 17.29)
			0.550	0.117
**Radio post**	2 (13.3%)	48 (15.3%)	0.85 (0.19, 3.88)	0.79 (0.15, 4.16)
			0.833	0.785
**Chemo pre**	2 (13.3%)	28 (8.9%)	1.57 (0.34, 7.29)	4.51 (0.72, 28.43)
			0.568	0.109
**Chemo post**	5 (33.3%)	88 (28.1%)	1.28 (0.42, 3.85)	1.36 (0.38, 4.86)
			0.662	0.635
**Hormone therapy**	11 (73.3%)	178 (57.1%)	2.07 (0.65, 6.64)	1.79 (0.51, 6.33)
			0.221	0.364
**Device**				
DTI	9 (60.0%)	183 (58.5%)	3.78 (1.31, 10.91)	2.66 (0.74, 9.51)
			0.014	0.132
TE	6 (40.0%)	130 (41.5%)	0.94 (0.33, 2.70)	0.71 (0.23, 2.24)
			0.906	0.561
**Mastectomy**				
Prophylactic	1 (6.7%)	60 (19.2%)	1.12 (0.21, 6.89)	4.02 (0.31, 24.41)
			0.779	0.057
Therapeutic	14 (93.3%)	253 (80.8%)	3.32 (0.43, 25.74)	2.88 (0.32, 26.10)
			0.251	0.348

### 3.2 The measure of health-related quality of life

213 patients adequately responded to the domains of the BREAST-Q questionnaire. A comparison between pre-operative and post-operative (2-year follow-up) self-reported scores was analyzed. Overall Satisfaction with Breasts, Psychosocial Well-being, and Sexual Well-being scores were all significantly increased after sub-cutaneous pre-pectoral DTI immediate reconstruction with TCPM mesh (*p* < 0.05) compared to CG. Patients scored a high level of Overall Satisfaction with Outcome index, measured postoperatively. The 15 patients who underwent prosthesis removal (either implant or TE) for wound dehiscence and infection reported lower post-operative scores compared to the average in the self-reported measures of Overall Satisfaction with Outcome (74.1), Overall Satisfaction with Breasts (69.1), Psychosocial Well-being (81.9), Sexual Well-being (63.1), and Physical Impact (79.2). The 15 patients scored a moderate increase from the baseline in all of the domains, except for Sexual Well-being and Physical Impact measures, which were decreased postoperatively. In any event, these changes were not statistically significant.

### 3.3 Clinical aesthetic outcomes

**Fig. 2:**
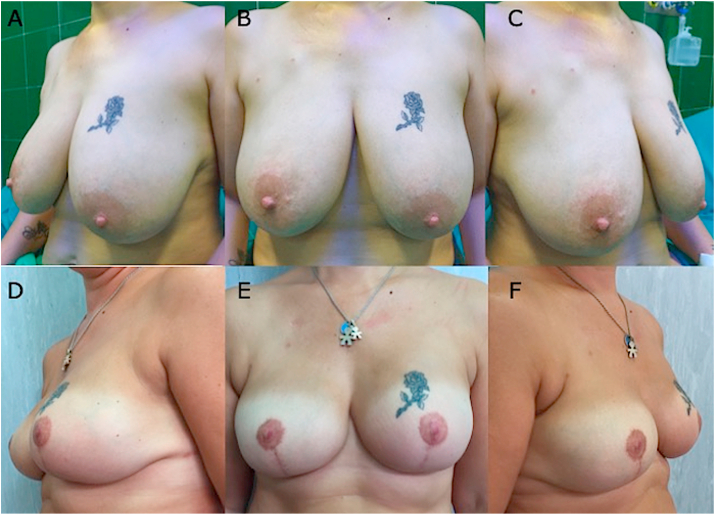
40 years old female patient who underwent bilateral SRM with Nipple Sparing, DTI immediate reconstruction positioning the prosthesis wrapped with TCPM in the pre-pectoral plane, and re-grafting of NAC according to Thorek technique (Intra-surgical view was shown in Fig. 1. A). 3/4 left pre-operative view. B) Pre-operative in frontal view. The breasts appear to be voluminous (macromastia) and with high-grade ptosis (pendulous breasts). C) 3/4 right pre-operative view. D) 3/4 left postoperative view after 2 years. E) Post-operative in frontal view, 2 years after the conservative mastectomy, pre-pectoral DTI, and TCPM mesh assisted reconstruction. The breast appears symmetric and with a good aesthetic outcome. F) 3/4 right post-operative view after the same time.

High scores, for breast volume, breast shape, position of infra-mammary fold, and scars were reported post-operatively. Mean satisfaction with the overall aesthetic result was high after breast reconstruction (8.72) in patients who underwent DTI pre-pectoral immediate reconstruction (SG) after 2 years from surgery (Fig. 2A–F). Aesthetic outcome scores are as follows: symmetry, 3.8; shape, 4.1; scars, 4.3; volume, 4.4; the position of the infra-mammary fold, 4.1; and overall evaluation score, 8.4.

### 3.4 Histological analysis

**Fig. 3:**
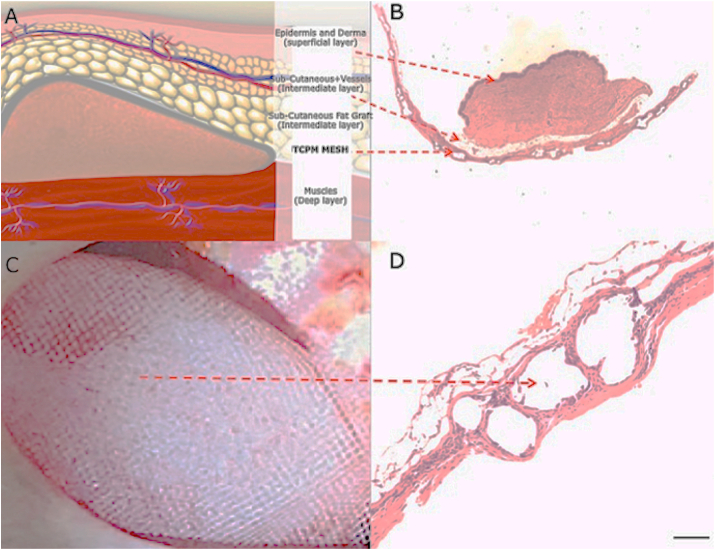
Histological analysis of TCPM mesh incorporation in breast soft tissue. A) Graphical reproduction of breast soft tissue anatomy, with focus on each layer; B) Hematoxylin-eosin image of post-operative biopsy of breast tissue involved in mesh graft; C) Capsule with TCPM mesh fully integrated; D) Magnification 4x inset of breast tissue directly harvested where the mesh was placed; collagen and fibroblast are identified, additionally, physiological restitutio ad integrum is observed.

**Fig. 4:**
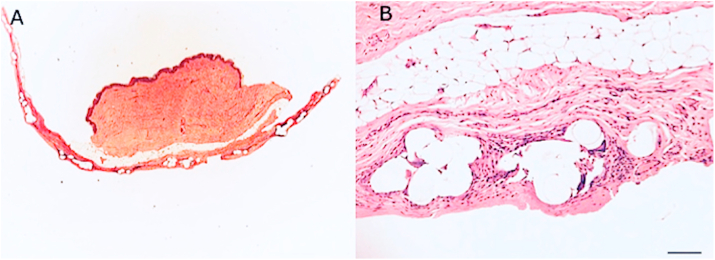
Histological analysis of TCPM mesh incorporation in breast soft tissue. A) Hematoxylin and eosin stain of post-operative punch biopsy of breast tissue involved in TCPM mesh graft; B) Magnification 4x inset of image A) in which it is possible to distinguish different layers containing collagen, abundant capillaries sprout, fibroblasts, and in particular the layer of adipocytes in which it will be possible to perform fat grafting to improve the volume and aesthetic outcomes. Complete healing and newly deposed dermal collagen.

As known, the breast tissue may be distinguished in several different layers, such as the epidermis and derma (superficial layer), the subcutaneous tissue containing fat and gland (intermediate layer), and muscles (deep layer) as graphically shown in Fig. 3A. These layers were displayed well in Hematoxylin-eosin staining of post-operative biopsies (Figs. 3B and 4A). 11 patients and related samples were selected with simple randomization (randomly). The allocation sequence has been created using an online randomization generator (https://www.randomizer.org) and has been concealed by a person unrelated to the trial management group.

In this series, tissue’ fragments incorporating TCPM mesh were analyzed (Fig. 3C). Collagen and fibroblast were identified, displaying complete incorporation of the mesh with physiological aspects of healing (Figs. 3D and 4B).

### 3.5 Bio-molecular assessment of mastectomy flap biopsies

**Fig. 5:**
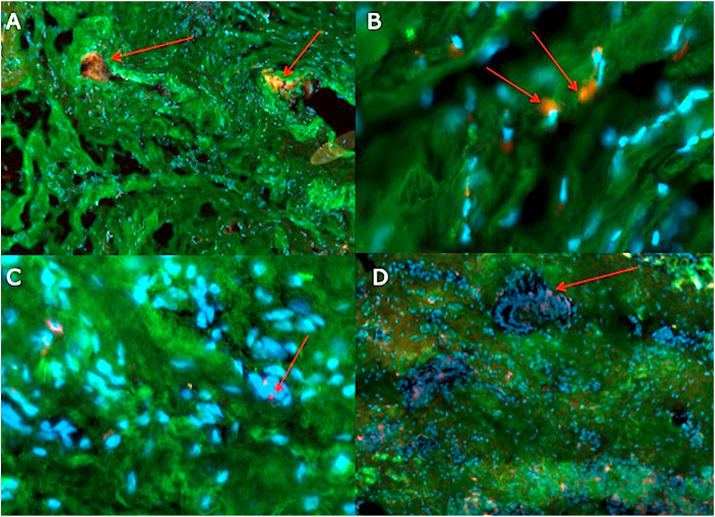
Double Immunofluorescence with CD 45 and Collagen 1. A) and B) images of TCPM mesh in a patient who underwent radiotherapy. The green color indicates collagen type 1, red color indicates Collagen type 3, with Nuclei contrasted in blue (DAPI staining). Red arrows indicated the lymphocytes. C) and D) immunofluorescence imaging of a patient who didn't undergo radiotherapy and TCPM use.

The immunofluorescence using CD 45+ and Collagen 1 (Fig. 5A–D), was performed in irradiated tissue treated with TCPM mesh (Fig. 5A and B) and in tissue that was not treated with radiotherapy and that was not reconstructed using TCPM (Fig. 5C and D). Fig. 5A and also Fig. 5B displayed a higher concentration of lymphocytes (red arrows) compared to Fig. 5C and D.

**Fig. 6:**
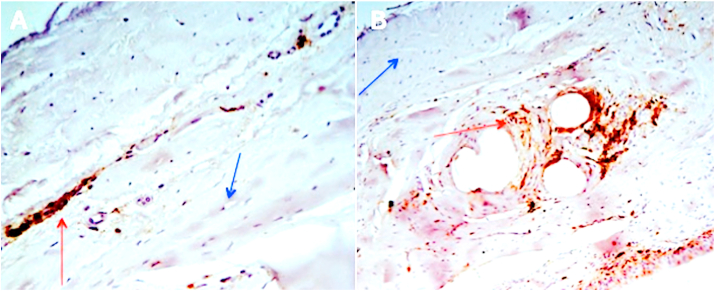
Immunohistochemistry using CD 45 on paraffin samples. A) 4x magnification and B) Imaging of TCPM mesh in a patient who underwent radiotherapy. Red arrows indicated the lymphocytes while blue arrows indicate fibroblasts.

The immunohistochemistry using CD 45+ on several paraffin samples displayed (Fig. 6A and B) the presence of lymphocytes (red arrows) and fibroblasts (blue arrows) around the TCPM mesh in irradiated tissue.

**Fig. 7:**
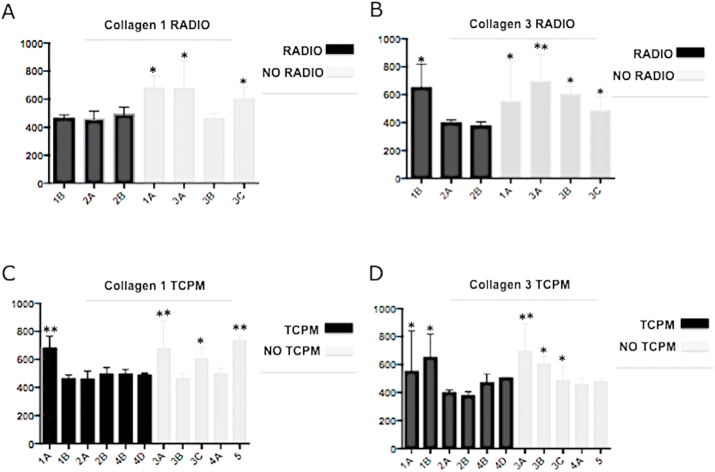
Bar graph analysis of Collagen 1 and 3 expressions. A) Collagen 1 expression in samples of who patients underwent radiotherapy and not; B) Collagen 3 expression in samples of patients who underwent radiotherapy and not; Collagen 1 expression in samples of patients who underwent TCPM mesh and not; Collagen 3 expression in samples of patients who underwent TCPM use and not. Collagen 1 and 3 expressions are higher in samples of patients who didn't undergo radiotherapy. The statistical symbols *, **, indicates the maximum value, where *, is the maximum value; **, are two values above the limit.

The bar graph displayed in Fig. 7A–D analyzes the differential expression of Collagen 1 (Fig. 7A and C) and Collagen 3 (Fig. 7B and 7D) concluding that Collagen 1 and 3 expression is the same (no statistical difference was observed) between TCPM and NO TCPM samples while is higher in samples of patients who didn't undergo radiotherapy (*p* < 0.05 vs. radio).

All the analyses performed confirmed the presence of inflammation in irradiated tissue as revealed by the high number of lymphocytes and also the complete integration of the TCPM mesh with physiological aspects of healing. In detail, the Collagen 1 and 3 expressions did not differ, with statistical significance, between TCPM and NO TCPM samples to confirm a process of healing demonstrating a perfect device incorporation and normal healing. Contrarily, the higher expression of Collagen 1 and 3 in tissue free from radiotherapy confirms the better quality of the subcutaneous layer of these tissues over radiotherapy tissues.

## 4 Discussion

Complications of conservative mastectomies with immediate implant-based reconstruction include infections, wound dehiscence, and implant loss, capsular contractures, asymmetries, and deformities with poor QoL results [22,23].

In patients who underwent NSMs, flap necrosis and NAC necrosis also may be considered relatively common complications. Headon et al. [24] conducted a pooled analysis of 12,358 NSMs to evaluate the adverse events and related oncological safety. The overall adverse events rate was 22.3% and the NAC necrosis rate was 5.9%. In this study, it is necessary to highlight that the rates of adverse events, including NAC necrosis, decreased over time, which was attributed to the improvement of the surgeon's expertise. They had reported that the factors predisposing to NAC necrosis were big and ptotic breasts, smoke, peri-areolar incision, and previous radiotherapy.

An investigation of the European Institute of Oncology [25] reported that comorbidity, smoke, incision type, flap thickness, and reconstruction's choice all influenced the NAC necrosis rate in NSMs.

PBBR is the most frequent reconstructive choice nowadays mostly in the case of conservative mastectomies like NSM [26,27]. In the early era of PBBR, sub-cutaneous prosthesis positioning represented the first approach, as reported by Snyderman in 1971 [28]. Subsequently, also the pioneers of TE, Radovan, and Lapin, adopted a sub-cutaneous positioning of their expanders [29,30]. By that time, however, most of the reconstructions were considered and performed in the setting of a previous radical mastectomy without preservation of pectoralis major muscle. Thanks to the development of modified radical mastectomy and to avoid some side effects of the sub-cutaneous procedures, like capsular contracture and prosthesis extrusion, during 1981, Gruber advocated the superiority of a sub-muscular (retro-pectoral) prosthesis reconstruction versus the Snyderman's procedure [31]. This important concept was also transferred to the TE approach performed by Argenta [32]. Nonetheless, a pre-pectoral sub-cutaneous approach was still claimed as the best option even later by Artz [33], when immediate two-steps PBBR was becoming quite popular. From this time (1988–1990), the sub-cutaneous technique eventually lost its initial appeal and for more than two decades almost disappeared as a choice of breast reconstruction. Implant coverage by a muscular pocket to interpose a viable cushion in case of skin flap/wound dehiscence and to allegedly prevent capsular contracture has represented the most commonly utilized choice for many years so far. Very few articles of sub-cutaneous positioned implants and TE were published in the early XXI century [34,35]. Recently, with the adoption of soft tissue replacement devices, either biological or synthetic, identified as “supportive materials”, the pure retro-pectoral trend has been modified, changing the choices in PBBR. A combined muscular-matrix pocket has become a very frequent option. The advantages offered by such devices were represented by an enlargement of the pocket and by the possibility to perform a definitive PBBR with an implant of right volume in a one-step surgery, so-called DTI.

The use of these supportive materials allows a better lower pole and infra-mammary fold tailoring not in DTI alone, but also in two-step TE reconstruction. During the last years, the introduction of these supportive materials has led to a completely novel approach consisting of a pre-pectoral prosthesis positioning, entirely covered by a biological or synthetic matrix, which is actually like “revisiting an old place” as several authors stated [36]. The rationale for a pre-pectoral reconstruction performed with these supportive materials is to replicate the muscular coverage offered by major pectoralis muscle during a retro-pectoral approach. Such devices, in fact, proved to be safe when positioned under the mastectomy cutaneous flap in the lower lateral pole, in the aforementioned dual-plane technique, and, hence, where mechanical stress forces are highest. Therefore, the idea of a full coverage extended to all prosthetic surfaces rapidly emerged. Thus, these devices may replicate, in a pre-pectoral and sub-cutaneous approach, the same coverage effect of muscles in the retro-pectoral technique, without any muscular detachment, offering an even more natural result. Additionally, it is necessary to highlight, that a pre-pectoral approach in PBBR is minimally invasive (very less invasive than the retro-pectoral approach), leaving all muscles intact and therefore allowing any future strategy change. Thanks to the fact that the muscles are intact, it will be possible, in fact, where necessary, to perform a sub-muscular reconstruction or even adopt an autologous flap option. Additionally, one of the greatest advantages of the pre-pectoral approach is the reduction of post-operative pain and the risk of animation deformity.

Nonetheless, in some cases, there is still some drawback to be faced in the pre-pectoral approach, which is, sometimes, an insufficient coverage thickness on the upper pole, avoided by pectoralis muscle, during the sub-muscular approach. This might lead to a visible prosthesis border and some moderate rippling on the upper profile (identified as “Wrinkling").

This drawback may be determined by the different thicknesses of supportive material versus major pectoralis muscle, and it may be more evident especially when the skin flap is very thin.

The study of Casella et al. [19] reported the immediate and long-term surgical and BREAST-Q results of 179 patients who underwent a mastectomy and immediate reconstruction with TiLoop® Bra mesh (comparable to TCPM). In this report, based on DTI muscle-sparing sub-cutaneous reconstruction, the prosthesis was entirely wrapped within a TCPM bag and positioned under the skin flap, following conservative mastectomy. The 2-year results of QoL, measured by the BREAST-Q questionnaire, confirmed high patients’ satisfaction following mastectomy and TiLoop® immediate reconstruction.

From another point of view, several concerns have been displayed by oncological-breast surgeons on the pre-pectoral approach and, in particular, on performing mastectomy with thick skin flaps, which could reduce the oncologic radicality of the procedure in an attempt to save as much more tissue as possible. On this issue, the authors of the present work in agreement with the data of the previous study published by Casella et al. [19] and others as Woo et al. [36], confirm that each mastectomy has to be performed following the plane of dissection at the level of Cooper's ligament (the only one correct plane of dissection in mastectomy procedures) removing all grossly visible breast tissue, including the sub-areolar breast tissue in case of NSM, aside from the thickness of skin flaps. Only this must be considered as an oncologically safe mastectomy procedure.

In the present work, the local tumor recurrence rate was 5.8% including three recurrences in the NAC (1,9%), two recurrences in axillary lymph-nodes (1,3%), three recurrences in skin flap (1,9%) and one recurrence (0,7%) in the muscle. In detail, the recurrence rate in the NAC (1,9%) appears to be in line with the literature and several important papers published by Galimberti et al. [37] and Lanitis et al. [2] reporting a recurrence rate in the NAC of 0–3.8%, and 0–3.7% respectively. On the other hand, a problem relating to the evaluation of “supportive materials”, especially about post-operative complication rates and proper patient selection is represented by the type of studies conducted, mainly represented by “single clinical trials” with a limited number of patients. Only recently Masià et al. [38] in a multicentric retrospective audit, collected the experience of 30 centers on pre-pectoral breast reconstruction with Braxton porcine ADM (Braxon; DECOmed Srl, Venice, Italy). A total of 1450 procedures, carried out by wrapping the implant with a pre-shaped porcine ADM, were retrospectively collected in a period of six years. In this study, diabetes, smoke, and immunosuppression had an influence on complications occurrence, as well as implant weight. Capsular contracture was associated with adjuvant radiotherapy, also if the overall percentage was low (2.1%). Complications led to implant loss in 6.5% of the cases. In agreement with the above-mentioned results, the study of Onesti et al. [39] confirmed the low rate of postoperative complications, using the same porcine ADM.

Additionally, there is still no consensus on whether synthetic matrices or biological matrices produce the best outcomes. In a review of Logan Ellis et al. [40], there were analyses of the differences in aesthetic outcomes, cost, and the rates of the most commonly reported complications. Here, the results display that TCPM synthetic mesh acts as bioactive material, producing breast reconstruction with remarkable aesthetic outcomes, with lower costs and complication rates.

The individual results for complication rates show that biological matrices are associated with lower infection rates and slightly lower capsular contracture, but higher hematoma rates, and slightly higher rates of skin necrosis and explanation—although many had post-op radiotherapy.

De Vita et al. [42] confirmed the absence of major side effects and excellent aesthetic outcomes performing IBR with pre-pectoral approach using polyurethane-coated implant, concluding this method represents a feasible alternative to subpectoral implant placement as confirmed also in very recent works published by Franceschini et al. [43] and Salgarello et al. [44]. On the other hand, it appears also pivotal analyze the cost of these procedures performing an economic evaluation.

A treatment’ economic evaluation (defined as a comparative analysis of alternative courses of action in terms of both their costs and consequences) primarily serves as a pragmatic aid to decision making [45]. Drummond et al. [45], highlights that the economic evaluation only addresses one dimension of treatment decision and that questions related to efficacy, effectiveness, and availability should be answered before an economic evaluation takes place.

An accurate “cost-analysis” of these treatments, based on supportive materials use, have been performed by several authors. In particular, Karp et al. [46], performed a retrospective review on all consecutive pre-pectoral one-stage breast reconstructions using ADM and synthetic mesh at a single institution. Several information as mastectomy type, reconstruction specs, results obtained, and supportive materials costs were analyzed. Calculated cost savings of synthetic mesh and ADM was up to $3415 in unilateral and $6830 in bilateral cases. Karp et al. [46], concluded the pre-pectoral breast reconstruction using ADM inferiorly and synthetic mesh superiorly was a safe technique that decreased material costs associated with support materials and allowed the plastic surgeon to precisely control the implant pocket and position.

Additionally, should be specified that DTI (one stage procedure) is more affordable than TE requiring two stages procedures.

## 5 Conclusions

Based on the data reported so far in this paper, it is possible to affirm from present study that conservative mastectomies as NSM and SRM are oncologically safe, provided that patients are carefully selected. These procedures when associated with immediate DTI reconstruction with the pre-pectoral approach and mesh use, not only preserve breast appearance immediately but also provides the opportunity for remodeling breast profile, possibly using fat graft besides, further enhancing women appearance. Concluding, six points must be highlighted:

1. Conservative mastectomies with pre-pectoral reconstruction must be considered oncologically safe in patients carefully selected;
2. The association of NSM and SRM with DTI reconstruction consents to have a single one-step surgery of conservative mastectomy and immediate reconstruction in large ptotic breasts;
3. Pre-pectoral sub-cutaneous DTI reconstruction is less invasive than retro-pectoral (sub-muscular) approach because the muscles are left intact;
4. The use of TCPM mesh in pre-pectoral sub-cutaneous DTI reconstruction is safe and replicates in many and selected cases a prosthesis coverage similar to the major pectoralis muscle, with distinguished self-reported QoL assessment scores.
5. The histological analysis of fragments of implant's wrapping tissue incorporating TCPM mesh displayed complete incorporation of the mesh with physiological aspects of healing.
6. The immunochemistry concluded the Collagen 1, and 3 expressions did not differ, with no statistical significance, between TCPM and NO TCPM samples, showing a process of physiological healing entailing a perfect device incorporation and confirming that TCPM is a bioactive material.

## Data availability

All data generated or analyzed during this study are included in this published article and its additional files.

## Funding statement

None.

## CRediT authorship contribution statement

**Pietro Gentile:** Conceptualization, Methodology, Software, Validation, Formal analysis, Data curation, Writing – original draft, Writing – review & editing, Visualization, Supervision, Project administration, Funding acquisition, conducted the study as leader. **Marco Bernini:** Methodology, Software, Formal analysis, Investigation, Data curation, Writing – review & editing, Visualization. **Lorenzo Orzalesi:** Software, Resources, Data curation, Investigation. **Silvia Sordi:** Formal analysis, Resources, Data curation. **Icro Meattini:** Data curation. **Francesca Lessi:** Formal analysis, Data curation. **Ashutosh Kothari:** Software. **Claudio Calabrese:** Conceptualization, Methodology, Software, Validation, Formal analysis, Investigation, Data curation, Tables .

## Declaration of competing interest

The authors declare that they have no known competing financial interests or personal relationships that could have appeared to influence the work reported in this paper.
